# The Treatment of Unfilled Root-Canals

**Published:** 1901-01

**Authors:** T. W. Onderdonk


### THE TREATMENT OF UNFILLED ROOT-CANALS.[1]

BY DR. T. W. ONDERDONK.

Mr. President and Gentlemen,—The subject I have chosen to-night is, The treatment of teeth which have been filled for a long time, and comfortable, but with unfilled root-canals.

I desire, first, to ask the society's pardon for bringing forward this subject. It is one, however, which is responsible for a great many mistakes on my part, and I wish to secure the opinion of as many as possible concerning the treatment of this class of teeth.

My treatment is as follows: For a clear comprehension we will make an arbitrary division: first, pericementitis; second, acute and chronic abscess; third, those disclosed by accident.

*Pericementitis.*—Not being sure of the history, as to whether the root is filled or not, I paint the gum with aconite and iodine, give the patient capsicum plasters (with instructions how to use them), also chloroform and listerine equal parts to apply to the sore spots on a piece of cottonoid. If this affords relief, I consider the treatment ended.

An acute abscess I consider only a second stage of pericementitis that has progressed too far to stop short of suppuration. My treatment is to evacuate the pus through an external opening, and when the soreness has subsided open the pulp-chamber, cleanse, disinfect, and close at once.

*Chronic Abscess.*—I open the pulp-chamber, cleanse the canal or canals, enlarge the apex sufficiently to allow the passage of disinfectants, thereby thoroughly cleansing the abscess tract.

*Those disclosed by Accident.*—When I say accident I mean those teeth which have new decay, broken enamel, or discolored teeth which we wish to crown, and when opened are found to have dead pulps. My remarks to-night apply particularly to these teeth. Here again we will make an arbitrary division,—first, when we find dry and gaseous conditions; second, creamy pus filling the pulp-chamber; third, watery conditions.

*First.*—When I open the pulp-cavity and find dry or gaseous conditions, I look for evidence of an old abscess. If found, I open the pulp-chamber and root-canals, thoroughly disinfect, and fill at

---

[1] Read before The New York Institute of Stomatology.

once, completing the operation at one sitting. If no evidence of abscess present, I treat same as next division.

*Second.*—Condition of creamy pus. I open into the pulp-chamber and canals, washing out the contents with warm water followed by hydrogen dioxide, sealing the cavity or not as seems best, being governed by the amount of soreness.

*Third.*—Watery conditions. Hang out the danger signal, as trouble must be expected. Open the pulp-chamber and wash out the contents, but do nothing that will close the apex at first sitting. I could quote any number of cases substantiating my practice, but the general plan as suggested by Dr. F. Milton Smith in his paper read before this society is the method I have followed for some time, with a few changes (one important one). Quoting from Dr. Smith: "In summing up he would say that he regards as essential to success in root treatment, first, the rubber dam; second, free direct access; third, thorough cleansing mechanically and antiseptically; fourth, getting the antiseptic through the root; fifth, perfectly filling the root, immediately upon getting an aseptic condition, with an antiseptic root filling; sixth, sufficient confidence in the method to insure thorough work and the minutest attention to details."

Now, I would indorse all of this most heartily except the fifth statement,—*i.e.*, the immediate filling upon securing an aseptic condition at the first sitting, as I understand. How are we to know that we have secured the desired condition? I would suggest, instead of the immediate permanent filling, the insertion of a root-treatment on cotton or paper points, completely filling the canals, sealing the cavity with a temporary filling, completing the operation at the next sitting if the tooth is comfortable, giving a second disinfection from the apex. At least, I feel that the safest way. As sometimes we are in need of securing relief, I know of no better way than by the removal of root-dressing. At all events, we secure the benefits of an immediate closure, and have at hand the means of easy relief in case of need.

I would denominate the presence or absence of pain the physical test. I wish to suggest (although it was not my intention to do so at this time) what I am pleased to call the scientific test,—*i.e.*, having a culture made from the cotton dressing, also from a fresh specimen taken after a thorough disinfection when we think the root is ready to fill, and, with the aid of the bacteriologist, *know*

when we have secured an aseptic root. The success of immediate root-closure is without doubt secured by the removal of all dead matter, thorough disinfection, and the introduction of an antiseptic root-filling in a demonstrated aseptic canal. In cleansing I use the Donaldson broach, sulphuric acid, and in favorable cases the engine reamer to clear the canals. In disinfection I rely on hot air, hydrogen dioxide, and iodoform in ether. In filling I prefer gutta-percha points for all large canals; for the smaller ones a mixture of zinc oxide, carbolic acid, and iodoform, forcing it into all recesses possible.

I want to say a word about gutta-percha points. I believe them to be the best root-filling if the canal is absolutely dry; if it is not dry, I think they are the worst thing that can be used. I dry the root by means of chloroform or ether, with iodoform in solution, then the hot point and hot air. I introduce a gutta-percha point a little smaller than the canal, the point being moistened with chloroform and having a bit of iodoform on the end; this is carried to the extreme end of the canal; again use the hot air and soften the gutta-percha to a creamy consistency, and with pressure force into place, and I believe that canal will be closed as well as can be done by hand of man. Supposing that a portion has passed the end of the root up into the jaw, I know of no material that would be retained as well.

I hope these remarks will be accepted in the same spirit which prompted me to prepare them,—in order that the young man just starting in practice may save himself trouble and his patients suffering by not attempting to fill all roots at the first sitting without reference to unfavorable conditions which may be present.

